# The Correspondence Between Executive Functioning and Academic Achievement Among Children with Prenatal Alcohol Exposure †

**DOI:** 10.3390/children12070842

**Published:** 2025-06-26

**Authors:** Kristene Cheung, Susan Doyle, Kylee Clayton, Ana Hanlon-Dearman, Jo Ann Unger, Caelan Budhoo, Alyssa Romaniuk

**Affiliations:** 1 Department of Clinical Health Psychology, University of Manitoba, Winnipeg, MB R3E 3N4, Canada; kclayton@hsc.mb.ca (K.C.); junger1@hsc.mb.ca (J.A.U.); 2 The Manitoba FASD Centre, SSCY Centre, Winnipeg, MB R3E 3G1, Canada; ahdearman@rccinc.ca; 3 The Children’s Hospital Research Institute of Manitoba (CHRIM), Winnipeg, MB R3E 3P4, Canada; 4 Newfoundland and Labrador Health Services, St. John’s, NL A1B 3V6, Canada; susan.doyle@nlhealthservices.ca; 5 Department of Pediatrics & Child Health, University of Manitoba, Winnipeg, MB R3E 3N4, Canada; 6 Department of Psychology, University of Manitoba, Winnipeg, MB R3T 2N2, Canada; budhooc@myumanitoba.ca (C.B.); romaniu3@myumanitoba.ca (A.R.)

**Keywords:** academic achievement, executive functioning, FASD, prenatal alcohol exposure

## Abstract

**Background/Objectives**: Canadian guidelines for diagnosing fetal alcohol spectrum disorder (FASD) strongly recommend using direct measures to assess brain domains whenever possible. Executive functioning, one of the brain domains assessed, can be measured using direct and indirect measures; however, research has found discrepancies between these two forms of assessment and has not examined this relationship using ratings from the newest version of one of the most commonly used indirect measure of executive functioning, the second version of the Behavior Rating Inventory of Executive Functioning (BRIEF2). Academic achievement may also help explain discrepancies between these forms of assessment, especially in indirect executive functioning skills at school, because many of the items on the BRIEF2 Teacher Form are related to school skills. This study aimed to examine the relationship between direct measures of executive functioning, indirect measures of executive functioning, and academic achievement. **Methods**: Charts of 74 children who completed an FASD diagnostic assessment in Canada were included in this study (61% males; 58% with FASD; *M_age_* = 11.77). Direct and indirect measures of executive functioning across settings and academic achievement were assessed. **Results**: Few correlations between corresponding BRIEF2 and direct measures of executive functioning were significantly associated. There were several significant correlations between academic achievement and (a) educator ratings on the BRIEF2 and (b) direct measures of executive functioning. None of the caregiver ratings on the BRIEF2 were significantly associated with academic achievement. **Conclusions**: The results suggest that academic performance is related to BRIEF2 ratings of executive functioning skills at school and direct measures of executive functioning. Aside from a few exceptions, direct and indirect measures of the same executive functioning skill were not correlated.

## 1. Introduction

Fetal Alcohol Spectrum Disorder (FASD) may be diagnosed when an individual has a confirmed history of prenatal alcohol exposure (PAE) and evidence of significant impairment in at least three of ten brain domains, assessed based on Canadian guidelines [1]. Executive functioning (EF) is one of the brain domains assessed when diagnosing FASD. Unlike the other domains, EF can be assessed using both direct (i.e., standardized assessment tools administered in a one-to-one assessment setting) and indirect measures (i.e., caregiver and educator ratings of a child’s functioning at home and school, respectively) [1]; however, the guidelines recommend that direct measures should be used to assess areas of functioning when possible. This recommendation is important considering that research continually demonstrates a discrepancy between these two measures among children with PAE [2,3] and FASD [2]. This discrepancy has been found across direct measures of EF, including subtests from the Delis–Kaplan Executive Function System (D-KEFS) [2,4], the NEuroPSYchological Assessment (Second Edition) [2], versions of the Wechsler Intelligence Scale for Children (WISC) [2,5,6], and the Wechsler Adult Intelligence Scale (Fourth Edition) (WAIS-IV) [2]. The findings of past research align with the concept of hot and cold executive functions, where hot executive functions are observed in situations that are motivationally and/or emotionally driven, and cold executive functions are observed in situations that are not highly influenced by motivation or emotions [7]. Researchers have suggested that direct and indirect measures of EF map onto cold and hot executive functions, respectively; therefore, this discrepancy would be expected given that the impact of motivation and emotions is more likely to arise in real-world situations than in an assessment session.

The current study aims to build on past research in three ways. First, the relationship between direct and indirect measures of EF among children with PAE or FASD has only been examined using caregiver [2,4,5,6,8,9,10] and teacher [3,9] ratings on the Behavior Rating Inventory of Executive Function (BRIEF) [11]; however, the second edition of the BRIEF (BRIEF2) [12] was published in 2015. Examining correspondence between direct and indirect measures using the most recent measures is imperative since most clinicians have switched to using the BRIEF2 in their practice. Further, revisions in the BRIEF2 were intended to improve the clinical and research utility of the measure [13,14]. For example, the BRIEF2 was re-standardized with a larger, more representative sample and included more concise scales. The BRIEF2 also consists of the Self-Monitoring and Task-Monitoring clinical scales, whereas the BRIEF included the Monitor clinical scale only [13,14]. Developments also included improving psychometric properties to improve clinical interpretation, adding an infrequency scale to assist in detecting unusual response styles, and enhancing correspondence between item content and order across Parent and Teacher Forms. A review of the BRIEF2 also noted a lack of information about the relationship between BRIEF2 scores and direct EF measures in any population at the time of their review [14].

Second, aside from one study that examined the association between direct and indirect EF skills among children with PAE with and without FASD [9], most research in this area has focused on children with PAE with FASD or categorized groups based on other FASD nomenclature (e.g., fetal alcohol syndrome or alcohol-related neurodevelopmental disorder). Understanding the relationship between direct and indirect measures of EF among children with PAE, regardless of FASD diagnostic status, is imperative because it has implications for clinical assessments and diagnostic and assessment recommendations. Although the general profile of caregiver ratings on the BRIEF seems to be consistent across groups (i.e., the Working Memory clinical scale and the Organization of Materials clinical scale yielded the highest and lowest mean *T*-scores, respectively) [2,4,10], research has highlighted differences in severity of caregiver ratings on the BRIEF across clinical samples of children with FASD [2,4,10]. Differences across these samples emphasize the importance of replicating studies, especially if the results are used for clinical purposes within one particular diagnostic clinic.

Third, past studies in this area commonly speculate that other factors could impact the relationship between ratings and tests of EF (e.g., child and family sociodemographic factors, parenting stress, sensory differences, and adaptive functioning), but none have explored these potential factors. One factor that has been questioned is academic achievement, as several of the questions on ratings of EF are related to or highly impacted by academic abilities (e.g., completing and starting assignments, bringing school-related documents home, and writing quality). Clinically, it is common for educator ratings on the BRIEF2 to be elevated when a child is also experiencing difficulties with academic skills. This relationship could also be bidirectional, where challenges with EF skills could impact academic achievement. Therefore, EF ratings may be affected by a child’s academic challenges rather than difficulties with EF skills.

The objectives of this exploratory study were to examine (1) group differences between direct EF skills, indirect EF skills, and academic achievement between children with and without FASD; (2) the relationship between direct measures of EF and ratings among children with PAE with and without FASD, using primary, optional, and error scores of tests (where applicable) and an indirect measure of EF, the BRIEF2; (3) the relationship between indirect measures of EF and academic achievement; and (4) the relationship between direct measures of EF and academic achievement.

## 2. Materials and Methods

Data were collected by conducting a retrospective chart review of school-aged children and adolescents with confirmed PAE who were seen for an FASD diagnostic assessment at one clinic. Referrals were typically received from families, medical professionals, social services (i.e., child and family services), and the education system. We only included charts with (a) both caregiver and educator ratings on the BRIEF2, (b) at least one direct measure of EF (i.e., a test score), (c) at least one academic achievement test score, and (d) FASD diagnostic status based on the Canadian diagnostic guidelines [1]. Charts that did not include all this information were excluded. Charts were not excluded due to co-occurring conditions. Previous studies have used subsets of this data [2,3].

The study included 74 charts of children and adolescents seen between January 2017 and March 2023 (*M_age_* = 11.77, *SD_age_* = 2.87, *range* = 7.58 to 17.67; 60.81% males). Some of the children and adolescents included in this study were assessed during the COVID-19 pandemic. Several modifications to the in-person assessment process were made during this time. For example, clinicians (and the child being assessed, if possible) wore medical-grade masks, a plexiglass divider was set up between the clinician and child, and stimulus book pages commonly touched by children were covered in page protectors for easy cleaning. A few children and adolescents were assessed virtually onsite, where the child was in the assessment room and the clinician was in the attached observation room. We also tried to reduce the amount of contact between clinicians and families by completing clinical interviews virtually or over the phone. Whether the testing was completed in person or virtually, extra care was taken to ensure that the modifications to the assessment process minimally impacted the validity of the results. Virtual assessments (also known as TelePsychology) were attempted during this time, but the data from these assessments were not included in the dataset due to concerns about validity.

Fifty-eight percent of the sample had PAE with FASD (*n* = 43; *M_age_* = 11.39, *SD_age_* = 2.80, *range* = 7.58 to 17.67) and 42% had PAE without FASD (*n* = 31; *M_age_* = 12.29, *SD_age_* = 2.93, *range* = 8.42 to 17.58). Additional socio-demographic information stratified by FASD diagnostic status is presented in Table 1.

### 2.1. Sociodemographic and Assessment Measures

Sociodemographic information included the child’s gender, living arrangement at the time of the assessment, and age at their psychology assessment appointment. Attention-deficit/hyperactivity disorder (ADHD) and intellectual disability (ID)/intellectual developmental disorder (IDD) diagnostic status were based on a previous diagnosis or a diagnosis made at the time of the FASD diagnostic assessment. Overall cognitive functioning was assessed as part of the routine FASD diagnostic assessment using the WISC, Fifth Edition (WISC-V) [15] or the WAIS-IV [16], and was presented as a Full-Scale Intelligence Quotient (FSIQ) or a General Ability Index (GAI; only calculated for the WISC-V). Both scores were presented as standard scores (*M* = 100, *SD* = 15), based on Canadian norms.

### 2.2. Direct Measures of Executive Functioning

EF was assessed using D-KEFS tests [17] that were routinely administered as part of an FASD diagnostic assessment based on previous research supporting test utility among children with PAE [18], examinees’ ability to meet pre-requisite criteria, and assessment material considerations due to clinical modifications made during the COVID-19 pandemic. Clinical modifications included adjusting the D-KEFS test battery to swap out tests that involve frequently handled and challenging to clean materials (e.g., the Sorting Test or the Tower Test) with tests that require less physical contact (e.g., Twenty Questions). Not all tests routinely administered as part of the FASD diagnostic assessment were included in the present study due to small sample sizes. Where applicable, primary and optional/error scores across conditions of the following tests were included in the present study: (1) Verbal Fluency Test (VFT), which measures verbal initiation, word generation, task-monitoring, self-monitoring, cognitive flexibility (i.e., shifting), and processing speed; (2) Color-Word Interference Test (CWIT), which assesses verbal inhibition and cognitive flexibility; and (3) Design Fluency Test, which measures motor speed, visual-perceptual skills, visual attention, and problem-solving, while adhering to task rules. For this study, we selected tests that measured the same type of EF skill on the BRIEF2 clinical scales. For example, scores from the CWIT were included because the BRIEF2 includes an Inhibit scale. Performance was based on American age-normed scaled scores (*M* = 10, *SD* = 3), where higher scores indicated a stronger performance or a lower error rate/greater accuracy. Canadian norms were not available.

Working memory subtests of the WISC-V and WAIS-IV were also included as direct measures of EF. Children between the ages of 6 and 16 years old completed the WISC-V, which included the Picture Span and Digit Span subtests. Adolescents aged 16 years and older typically completed the WAIS-IV, which includes the Digit Span subtest (see Table 2 for sample sizes); however, some 16-year-olds completed the WISC-V. Test scores were presented as scaled scores based on Canadian norms (*M* = 10, *SD* = 3).

### 2.3. Ratings of Executive Functioning

We used the BRIEF2 Parent Form and Teacher Form [12] to measure ratings of EF among children and adolescents. The primary caregiver with whom the child lived at the time of the assessment often completed the Parent Form (see Table 1 for more details on their relationship with the child being assessed). Schools were encouraged to have the person most knowledgeable about the child complete the BRIEF2 Teacher Forms, which was often the child’s classroom teacher and, in some cases, the resource or guidance teacher. Therefore, we have used the phrase educator ratings instead of teacher ratings throughout this study. The BRIEF2 Forms consist of 63 items, ranked on a 3-point Likert scale, including *Never*, *Sometimes*, and *Often.* Responses created nine clinical scales: Inhibit, Self-Monitor, Shift, Emotional Control, Initiate, Working Memory, Plan/Organize, Task-Monitor, and Organization of Materials. Clinical scales combine to form one overall score (Global Executive Composite, GEC) and three first-order index scores: Behavior Regulation Index (BRI), Emotion Regulation Index (ERI), and Cognitive Regulation Index (CRI). Scores on the BRIEF2 were presented as age-based norms, using *T* scores (*M* = 50, *SD* = 10), where higher scores reflected more difficulties. *T*-scores were classified into three descriptive categories: (a) mildly elevated (*T*-scores = 60 to 64), (b) potentially clinically elevated (*T*-scores = 65 to 69), and (c) clinically elevated (*T*-scores ≥ 70). We excluded cases when data for more than one clinical scale was missing because we did not want to underpower the sample further. One of the caregiver ratings was missing a value for the Shift scale, and another was missing a value for the Task–Monitor scale.

### 2.4. Academic Achievement

The Wechsler Individual Achievement Test 3rd Edition (WIAT-III) [19] was used to measure academic achievement in children and adolescents aged 4 years, 0 months to 19 years, and 11 months. The following subtests are routinely assessed as part of the FASD diagnostic assessment and included as variables in this study: Word Reading, Pseudoword Decoding, Reading Comprehension, Spelling, Math Problem Solving, and Numerical Operations. Scores were presented as standard scores (*M* = 100, *SD* = 15) based on Canadian norms.

### 2.5. Statistical Analyses

We used the IBM SPSS^®^ software package (Version 27.0) for statistical analyses. First, we used descriptive statistics to calculate the sociodemographic and diagnostic characteristics, the percentage of children falling in the clinically elevated range for BRIEF2 clinical scales, and index and composite scores. Second, sample means, standard deviations, range of child age, overall cognitive functioning, and scores on direct and indirect measures of EF were also calculated. Third, we conducted independent *t*-tests to examine differences between test scores and ratings between children with and without FASD. Levene’s Test for Equality of Variances was conducted to determine which test statistics should be used based on whether equal variances were assumed. Fourth, zero-order correlations were performed between variables of interest. We used Bonferroni correction across the correlational analyses to account for multiple comparisons and reduce the increased risk of a Type I error. The adjusted *p*-values were determined by dividing a *p*-value of 0.05 by the number of correlations to be conducted. Results at *p* < 0.05 were considered statistically significant. Using G*Power 3.1.1. Software, with an alpha level of 0.05, a desired power of 0.8, and a medium effect size of *r* = 0.3, a minimum sample size of 84 was required. Sample sizes for each correlation are provided in the tables. Correlations were interpreted based on Cohen’s guidelines: small, *r* = 0.10 or *d* = 0.20; medium, *r* = 0.30 or *d* = 0.50; and large, *r* = 0.50 or *d* = 0.80.

## 3. Results

Table 2 presents the descriptive statistics of the EF test scores across the two groups. The performance across most tests among both children with and without FASD was below the normative mean (i.e., scaled score = 10 or index score = 100). There were several significant differences in performance between children with FASD and children without FASD, where children with FASD scored significantly lower for many of the D-KEFS tests and all the WIAT-III subtests. Supplemental Table S1 presents the descriptive statistics for BRIEF2 caregiver and educator ratings and the validity scales. All the mean caregiver and educator ratings for children with and without FASD were above the normative average. BRIEF2 caregiver and educator ratings stratified by FASD diagnostic status are provided in Supplemental Tables S2 and S3, respectively. The sample means for two BRIEF2 clinical scales (i.e., Inhibit and Task-Monitor) for caregiver ratings were significantly higher among children with FASD than without FASD. There were no significant group differences in educator ratings between children and adolescents with and without FASD.

Table 3 provides the zero-order correlations between BRIEF 2 caregiver and educator ratings and performance on tests of EF. A small to medium relationship was found for five correlations. For caregiver ratings, only the CWIT Inhibition/Switching: Total Errors (*r* = −0.31) was significantly correlated after corrections. For educator ratings: the Inhibit scale from the BRIEF2 and CWIT Inhibition: Total Errors (*r* = −0.38) and CWIT Inhibition/Switching: Total Errors (*r* = −0.35); Initiate scale and VFT Letter Fluency: Total Correct (*r* = −0.39) were also significantly correlated. See Supplemental Table S4 for correlations between direct measures of EF and BRIEF2 index scales and composite scores.

There was a small but non-significant relationship between caregiver ratings and academic achievement scores on the WIAT-III (see Table 4). Before Bonferroni corrections, there was a small to medium relationship between BRIEF2 educator ratings and academic achievement scores on the WIAT-III (see Table 5). Over half of the correlations were no longer significant after Bonferroni corrections, including any correlations between Pseudoword Decoding, Math Problem Solving, Numerical Operations, and BRIEF2 Clinical Scales. None of the WIAT-III subtests were significantly associated with ERI. Table 6 provides the zero-order correlations between direct measures of EF skills and academic achievement. Several significant correlations were found between tests assessing inhibition, initiation, and shifting and WIAT-III subtests before and after Bonferroni correction. Just under half of the correlations were no longer significant after Bonferroni corrections. None of the tests for self-monitoring or task-monitoring (after Bonferroni correction) were associated with WIAT-III subtests.

## 4. Discussion

Overall, this study showed that direct and indirect measures of EF skills that assess the same type of skills were not significantly correlated, with a few exceptions for teacher ratings. This is consistent with previous research with both clinical and non-clinical groups [2,3,4,5,6,9,10,20], suggesting that these findings result from differences in the measures versus being unique to the population studied here. Interestingly, some correlations between the direct EF measures were associated with the BRIEF2 in the current study but not with the BRIEF in previous studies [2,3]. For example, the association between teacher ratings of the BRIEF2 Initiate Scale and VFT Letter Fluency: Total Correct was significant in this study (*r* = −0.39, *p* < 0.01, *n* = 71) but not when assessed using the BRIEF (*r* = 0.1, *n* = 69 to *r* = −0.15, *n* = 96). Some of the teacher ratings of EF skills remained significantly related to academic skills after Bonferroni corrections, whereas none of the caregiver ratings of EF skills showed significant relationships. Some direct measures of EF skills were also significantly correlated with academic skills.

Consistent with past research, children with PAE without FASD generally performed better on EF tests than children with FASD [2,18,21]. This finding provides additional support for administering EF tests for diagnostic purposes. We also found that ratings on the BRIEF2 across groups fell within the potentially clinically elevated to the clinically elevated range, which aligns with previous studies [2,4]. This finding is not surprising considering the high rate of ADHD across the two groups, as children with ADHD tend to have elevated ratings on the BRIEF2. Overall, the discrepancy between direct and indirect measures of EF demonstrated in this study was consistent with previous studies [2,3,4,5,6,8,9,10]. Reviewing the definition of the different measures clarifies why the BRIEF2 scales failed to correlate with their respective direct measures. For example, self-monitoring is “the ability to monitor and evaluate your own performance” [22]. The self-monitor scale on the BRIEF2 assesses an individual’s awareness of how their behavior impacts others, whereas direct measures of self-monitoring, as defined in this study, refer to the child’s ability to monitor their behavior, such that they do not repeat previously stated answers.

We also found that some of the distributions of the scores for academic achievement tests differed between groups, while the distributions of the BRIEF2 measures were consistently similar. Taken together, these findings further suggest that EF skills could be distinguished as direct measures of EF skills based on test performance and indirect measures of EF or day-to-day EF skills based on informant ratings, as suggested by Gross et al. [5] and Mohamed et al. [4]. Additionally, EF skills may continue to be differentiated in terms of hot (situations that are motivationally and/or emotionally driven) versus cold (situations that are not highly influenced by motivation or emotions) executive functions [7]. This distinction could also explain how children and adolescents with PAE and FASD generally obtain lower scores on direct measures of EF than their counterparts, while both groups have high and similar ratings of EF. Children and adolescents with PAE often have difficulty with day-to-day or hot executive functions regardless of FASD diagnosis. In contrast, those diagnosed with FASD have more difficulties with performance-based or cold executive functions than their counterparts. Mohamed et al. [4] highlighted that the BRIEF overlaps with other areas of functioning, such as sensory processing, and other disorders, such as ADHD and autism spectrum disorder. They also suggested that the BRIEF, created to complement direct measures of EF, should continue to be used as a complementary measure. In contrast, direct measures like the D-KEFS are more helpful in examining cognitive deficits.

This study also demonstrated that several scores on tests of academic achievement were significantly associated with BRIEF2 results when assessed by educators but not caregivers. This finding could be related to the fact that educator ratings of EF skills are based on observations in the academic setting, where a child may not feel successful if they are struggling with academics. Indeed, there is a potential measurement overlap between the BRIEF2 academic-related items and the WIAT-III, which may inflate the observed correlations in the educator forms. In contrast, caregiver ratings of EF skills are observed within the home setting, where behaviors may or may not be impacted by academic abilities. Rather than being viewed as a limitation or inconsistency, discrepancies between BRIEF2 caregiver and educator ratings may provide valuable ecological insight into the contextual variability of EF skills. In turn, discrepant profiles between caregiver and educator BRIEF2 ratings may not indicate psychometric weakness or assessment inconsistency but rather provide critical ecological insight into how EF skills manifest differently across environments with distinct expectations, demands, and support structures. Children with PAE may demonstrate variable executive performance depending on contextual affordances—for example, the level of environmental structure, external prompting, or demands for self-initiation. Educators may observe greater EF challenges due to the academic complexity, behavioral expectations, and multitasking requirements inherent to classroom settings. In contrast, caregivers may observe relatively fewer difficulties at home, where routines may be more predictable, demands are personalized, and consequences are more immediate and relational.

Taken together with the discussion above, this suggests that day-to-day EF skills at school are more likely to correlate with academic achievement tests than direct measures of EF skills, at least based on the measures used in this study, which are routinely used in our diagnostic clinic. This finding is also important for clinicians to consider if they struggle to explain differences in ratings across settings. Interestingly, some BRIEF2 clinical scales were more likely to correspond to some academic skills than other clinical scales. For example, the Task–Monitor scale, was significantly associated with three of the six academic subtests (all related to literacy) after corrections. This scale assesses a child’s ability to monitor what they are doing, ensuring they complete the task correctly and neatly. A child who struggles with academics is likely to make more mistakes in their academic work and/or have difficulty completing their assignments. Several direct measures of EF skills in the domains of Inhibit, Initiate, Shift, and Working Memory were significantly related to academic achievement tests. There did not seem to be a consistent pattern of verbal versus visual-based direct measures of EF being more consistently associated with literacy skills versus mathematics, respectively; instead, these EF skills seem to be related to academic achievement more broadly. The only exception was the VFT Letter Fluency: Total Correct score, being significantly associated with literacy skills (reading and spelling) but not math skills. The D-KEFS manual highlights how this test can be impacted by reading and spelling abilities [17]. Previous research in the area of EF and academic achievement in children and adolescents with and without learning disabilities has shown that performance on tasks on Inhibition and Working Memory is consistently related to performance in reading and mathematics [23]. Previous research has also suggested that although the content of reading versus math-related academic tasks is quite different, EF performance tasks measure common cognitive processes applicable across academic domains [23]. Direct measures of EF skills of self- and task-monitoring appear to be less related to performance on academic achievement tests. These findings could support the interpretation of clinical findings in a diagnostic setting.

### 4.1. Clinical Implications

Regardless of which concept is used to explain potential discrepancies between direct and indirect measures of EF, the results of this study emphasize the importance of differentiating between the skillsets when providing assessment results and recommendations to caregivers and educators of children with PAE. Clinicians should also be mindful of how caregivers and educators may perceive seemingly inconsistent assessment results based on direct and indirect measures. For example, difficulties with initiation at home that are not captured by tests of initiation may leave a caregiver feeling invalidated or a child being misunderstood. Highlighting the difference between these two EF skills in different contexts for the same skills may open discussions around potential reasons for the discrepancy, such as identifying possible factors that may interfere with a child’s ability to apply a specific skill in a particular environment. Clinicians may also highlight the link between indirect measures of EF at school and academic achievement and/or discuss discrepancies between direct and indirect measures of EF when providing assessment feedback. For example, clinicians could acknowledge the differences between how direct and indirect measures are assessed, one being in a one-to-one, fairly distraction-free setting (direct measures) and the other being assessed in the real world with many competing demands (indirect measures). Along with previous research, the outcomes of this study further highlight the necessity of assessing both direct and indirect measures of EF as part of an FASD diagnostic assessment, especially if a child experiences difficulties with academics. The findings from this study may also dissuade clinicians from generalizing assessment data from one form of measurement to the other, as this could result in an overdiagnosis of FASD. In other words, ratings of day-to-day EF skills should not be substituted for tests of EF as part of an FASD diagnostic assessment when possible. The results of this study also offer additional support for using direct measures of EF for diagnostic differentiation, given that BRIEF2 ratings do not seem to differentiate between children with and without FASD.

### 4.2. Strengths and Limitations

The present study has several notable strengths. First, using the BRIEF2 allowed us to examine the relationship between an indirect and direct measure of EF using the most recent version of the BRIEF. This is helpful for clinical purposes, given that there are differences between the two versions, and most clinicians will or have started using the BRIEF2. Second, including optional and error scores offered alternative direct measures of EF and allowed for a more in-depth examination of the relationship of interest. Third, we included children with PAE with and without FASD in our study, whereas most studies have only included children diagnosed with FASD. Including children with PAE who are not diagnosed with FASD in research is especially valuable considering that about half of the children assessed for FASD are not ultimately diagnosed with FASD [2,3,24]. Understanding a child’s unique neurocognitive profile is important for post-assessment recommendations and intervention planning, regardless of diagnostic outcome. Including children with PAE without FASD in research also helps to assess the diagnostic validity of the measures used in the assessment. More variability in the analyses may also reduce the likelihood of the analyses being impacted by issues like restricted range.

The current research results should be considered within the following limitations. First, the results of this study may not be generalizable to all children with PAE, as only those referred and assessed at our clinic were included in this sample. Our clinic only sees children with notable concerns, meaning this study did not capture children with PAE without concerns at home or school. Further, FASD diagnostic status as a dichotomous variable may not accurately reflect the severity of impairment or functioning. Second, given that most of the children in this sample were cared for by foster parents at the time of referral, the level of familiarity of the child likely varied across caregivers. Third, as we only included measures administered as part of our routine assessment, other factors not examined in the present study could moderate or mediate the relationships examined in this study, such as informant stress, familiarity with the child, how long the informant has known the child, and performance-based variables (e.g., effort, motivation, and attention). Other factors like child age could also moderate or mediate the relationship. Fourth, the present study does not include a control group of children without PAE. Fifth, the results were based on cross-sectional, correlational data; therefore, causal inferences and directionality cannot be made. Sixth, shared method variance may be a potential source of correlation between educator BRIEF2 and WIAT-III scores. Seventh, our exclusion criteria may have introduced a bias toward less severe cases. Children not attending school at the time of the assessment were excluded because their charts did not include BRIEF2 teacher ratings. Additionally, our exclusion criteria limited the sample to families who completed the diagnostic process. Some families may begin the FASD diagnostic assessment process but do not complete it for various reasons and will, therefore, not have a diagnostic outcome. Eighth, we included caregiver and teacher ratings even if the validity scales were not in the “acceptable” range. These indicators are often elevated in the diagnostic clinic and may represent the true degree of difficulty experienced by the child or a nuanced interpretation of the questions [25]. Including protocols with elevated or highly elevated negativity validity scales may further reduce the likelihood of direct and indirect EF measures being correlated. Finally, unfortunately, our sample size is slightly underpowered based on the required 84; however, we used the charts with the available data, and increasing the sample size was not possible at the time of the analysis. Therefore, it is important to interpret the results cautiously.

### 4.3. Directions for Future Research

Future researchers should continue examining the association between direct and indirect measures of EF by using different assessment measures, especially those typically used as part of a clinic’s routine test battery, such as the NEPSY-II or the Comprehensive Executive Function Inventory (CEFI). Alternatively, considering some notable differences in the severity of difficulties between clinic samples [2,4,10], and to enhance power, researchers should replicate previous studies and examine this relationship using the same assessment tools reviewed in the current study. Future studies should also attempt to explore potential moderators or mediators on the strength and significance of the relationship between direct and indirect measures of EF, including those suggested above. Finally, intervention studies on the utility of conceptualizing EF in terms of skill performance and application, as indicated by Gross et al. [5], should be conducted to determine the feasibility and effectiveness of such programming. Considering the limitations of the cross-sectional design of this study, future researchers could conduct a longitudinal analysis to determine the developmental trajectories of EF and academic achievement in children with PAE. Given that this study was underpowered, future research should replicate it with the required sample size and consider stratifying the sample by FASD diagnostic status.

## Figures and Tables

**Table 1:** Socio-demographic and diagnostic variables among children and adolescents with prenatal alcohol exposure.

	Total Sample%	PAE with FASD%	PAE Without FASD%
**Gender**			
Female	39.19	39.53	38.71
Male	60.81	60.47	61.29
**Living Situation**			
Birth family	16.22	13.95	19.35
Foster care	43.24	44.19	41.94
Adoptive family	6.76	11.63	0.00
Extended family	33.78	30.23	38.71
**ADHD Diagnosis**			
No	36.49	34.88	38.71
Yes	63.51	65.12	61.29
**ID/IDD Diagnosis**			
No	83.78	72.09	100.00
Yes	16.22	27.91	0.00

Note. ADHD = attention-deficit/hyperactivity disorder; FASD = fetal alcohol spectrum disorder; ID/IDD = intellectual disability/intellectual developmental disorder; PAE = prenatal alcohol exposure.

**Table 2:** Assessment data variables among children and adolescents with prenatal alcohol exposure.

	PAE with FASD			PAE Without FASD				
	*n*	*M* (*SD*)	*Range*	*n*	*M* (*SD*)	*Range*		
**Inhibit**							*t* (95% CI)	*d*
CWIT I: CT	40	7.43 (3.40)	1–13	29	8.76 (2.31)	4–13	1.94 (–0.04, 2.71)	0.45
CWIT I: TE	37	7.08 (3.45)	1–13	28	9.14 (2.70)	4–14	2.61 * (0.48, 3.64)	0.65
CWIT I/S: CT	38	7.82 (3.19)	1–15	29	8.83 (3.01)	3–13	1.32 (−0.52,2.54)	0.33
CWIT I/S: TE	35	6.29 (3.78)	1–11	28	8.93 (2.73)	3–13	3.22 ** (1.00, 4.29)	0.79
**Initiate**								
VFT LF: TC	42	6.69 (3.00)	1–18	29	9.00 (3.49)	3–17	2.98 ** (0.77, 3.85)	0.72
VFT CF: TC	42	7.36 (3.01)	1–14	29	9.41 (3.32)	4–18	2.71 ** (0.54, 3.57)	0.66
VFT CS: TC	42	7.33 (2.79)	1–15	28	9.61 (2.41)	6–14	3.52 ** (0.99, 3.56)	0.86
DF TAD	23	10.43 (3.96)	4–18	19	10.21 (3.46)	6–17	−0.19 (−2.57, 2.12)	−0.06
**Self-Monitor**								
VFT RE	40	8.20 (2.83)	1–12	28	8.71 (2.29)	1–12	0.80 (−0.78, 1.80)	0.20
VFT Percent RE	40	8.13 (4.26)	1–12	28	9.21 (3.41)	1–13	1.17 (−0.77, 2.95)	0.28
DF TRD	24	9.54 (3.36)	2–13	20	11.40 (1.98)	5–13	2.27 * (0.20, 3.51)	0.66
**Shift**								
VFT CS: SA	42	7.02 (3.10)	1–14	29	9.10 (2.83)	3–15	2.88 ** (0.64, 3.52)	0.70
VFT CS: Percent SA	40	6.88 (4.11)	1–12	28	8.32 (3.73)	1–12	1.48 (−0.50, 3.39)	0.37
CWIT I/S: CT	38	7.82 (3.19)	1–15	29	8.83 (3.01)	3–13	1.32 (−0.52, 2.54)	0.33
CWIT I/S: TE	35	6.29 (3.78)	1–11	28	8.93 (2.73)	3–13	3.22 ** (1.00, 4.29)	0.79
DF Switching: TC	24	6.96 (3.48)	2–19	20	9.30 (2.18)	5–12	2.61 * (0.53, 4.15)	0.79
**Task-Monitor**								
VFT SLE	40	8.70 (4.00)	1–13	28	9.93 (2.84)	4–13	1.40 (−0.52, 2.98)	0.35
VFT Percent SLE	40	7.48 (4.81)	1–14	28	9.11 (4.30)	1–14	1.44 (−0.64, 3.90)	0.35
DF TSD	24	7.71 (4.40)	1–14	20	9.80 (3.76)	1–14	1.68 (−0.43, 4.61)	0.51
**Working Memory**								
Digit Span	33	5.76 (1.94)	2–10	24	7.29 (1.83)	4–13	3.02 ** (0.52, 2.55)	0.81
Picture Span	28	7.32 (2.39)	3–11	21	9.29 (3.17)	5–17	2.48 * (0.37, 3.56)	0.72
**Cognitive** **Functioning**								
FSIQ	34	73.38 (8.03)	58–94	26	86.88 (9.56)	72–119	5.94 *** (8.96, 18.05)	1.55
GAI	29	75.41 (9.32)	57–98	21	91.24 (10.27)	74–118	5.68 *** (10.22, 21.43)	1.63
**Academic** **Achievement**								
PD	34	76.53 (16.36)	46–107	28	87.68 (16.72)	58–119	2.64 * (2.72, 19.58)	0.68
WR	35	80.00 (15.19)	53–117	29	88.52 (16.75)	57–117	2.13 * (0.53, 16.51)	0.54
RC	28	75.79 (11.82)	45–105	26	87.35 (11.81)	68–126	3.59 ** (5.10, 18.02)	0.98
Spelling	33	76.18 (11.62)	53–94	29	86.03 (12.51)	67–114	3.22 ** (3.72, 15.98)	0.82
NO	35	74.14 (8.92)	53–95	30	81.03 (10.07)	64–105	2.93 ** (2.18, 11.60)	0.73
MPS	35	71.06 (10.39)	40–93	28	82.43 (11.61)	68–116	4.10 *** (5.82, 16.92)	1.04

Note. 95% CI = 95% confidence intervals; CT = Completion Time; CWIT I = Color-Word Interference Test Inhibition; CWIT I/S = Color-Word Interference Test Inhibition/Switching; DF = Design Fluency; FASD = fetal alcohol spectrum disorder; FSIQ = Full Scale Intelligence Quotient; GAI = General Ability Index; MPS = Math Problem Solving; NO = Numerical Operations; PAE = prenatal alcohol exposure; PD = Pseudoword Decoding; RC = Reading Comprehension; RE = Repetition Errors; SA = Switching Accuracy; SLE = Set-Loss Errors; TAD = Total Attempted Designs; TC = Total Correct; TE = Total Errors; TRD = Total Repeated Designs; TSD = Total Set-Loss Designs; VFT CF = Verbal Fluency Test Category Fluency; VFT CS = Verbal Fluency Test Category Switching; VFT LF = Verbal Fluency Test Letter Fluency; WR = Word Reading. * *p* < 0.05. ** *p* < 0.01. *** *p* < 0.001.

**Table 3:** Correlations between corresponding direct measures of executive functioning and BRIEF2 scales.

	BRIEF2 Scales	Caregiver	Educator
**Tests of Inhibition**			
CWIT I: CT	Inhibit	0.11 (69)	−0.15 (69)
CWIT I: TE	Inhibit	−0.30 *^a^ (65)	−0.38 ** (65)
CWIT I/S: CT	Inhibit	0.14 (67)	0.01 (67)
CWIT I/S: TE	Inhibit	−0.31 * (63)	−0.35 ** (63)
**Tests of Initiation**			
VFT LF: TC	Initiate	0.13 (71)	−0.39 ** (71)
VFT CF: TC	Initiate	−0.03 (71)	−0.22 (71)
VFT CS: TC	Initiate	0.03 (70)	−0.09 (70)
DF TAD	Initiate	0.13 (42)	−0.18 (42)
**Tests of Self-Monitoring Skills**			
VFT RE	Self-Monitor	−0.02 (68)	−0.21 (68)
VFT Percent RE	Self-Monitor	0.05 (68)	−0.23 (68)
DF TRD	Self-Monitor	−0.10 (44)	−0.01 (44)
**Tests of Shifting/Switching**			
VFT CS: Total SA	Shift	−0.13 (70)	−0.11 (71)
VFT CS: Percent SA	Shift	−0.15 (67)	−0.07 (68)
CWIT I/S: CT	Shift	0.13 (66)	0.02 (67)
CWIT I/S: TE	Shift	−0.05 (62)	−0.14 (63)
**Tests of Task-Monitoring Skills**			
VFT SLE	Task-Monitor	−0.02 (67)	−0.12 (67)
VFT Percent SLE	Task-Monitor	0.03 (67)	−0.16 (67)
DF TSD	Task-Monitor	−0.23 (43)	−0.12 (43)
**Tests of Working Memory**			
Digit Span	Working Memory	0.22 (57)	−0.14 (57)
Picture Span	Working Memory	−0.03 (49)	−0.24 (49)

Note. Sample sizes are provided in brackets. CT = Completion Time; CWIT I = Color-Word Interference Test Inhibition; CWIT I/S = Color-Word Interference Test Inhibition/Switching; DF = Design Fluency; RE = Repetition Errors; SA = Switching Accuracy; SLE = Set-Loss Errors; TAD = Total Attempted Designs; TC = Total Correct; TE = Total Errors; TRD = Total Repeated Designs; TSD = Total Set-Loss Designs; VFT CF = Verbal Fluency Test Category Fluency; VFT CS = Verbal Fluency Test Category Switching; VFT LF = Verbal Fluency Test Letter Fluency. * *p* < 0.05. ** *p* < 0.01. ^a^ No longer significant after Bonferroni corrections with adjusted critical *p*-values.

**Table 4:** Correlations between BRIEF2 caregiver ratings and academic achievement.

	WIAT–III *r* (*n*)					
	PD	WR	RC	Spelling	MPS	NO
**Clinical Scales**						
Inhibit	−0.02 (62)	−0.02 (64)	−0.18 (54)	−0.11 (62)	−0.08 (63)	−0.06 (65)
Self-Monitor	0.18 (62)	0.05 (64)	−0.02 (54)	−0.001(62)	−0.09 (63)	−0.04 (65)
Shift	0.09 (61)	−0.02 (63)	0.20 (53)	0.05 (61)	−0.12 (62)	0.11 (64)
Emotional Control	0.12 (62)	0.08 (64)	0.19 (54)	0.04 (62)	−0.03 (63)	0.04 (65)
Initiate	0.23 (62)	0.08 (64)	0.22 (54)	0.05 (62)	−0.07 (63)	−0.04 (65)
Working Memory	0.12 (62)	−0.02 (64)	−0.01 (54)	−0.05 (62)	−0.09 (63)	−0.08 (65)
Plan/Organize	0.04 (62)	−0.09 (64)	−0.02 (54)	−0.15 (62)	−0.18 (63)	−0.11 (65)
Task–Monitor	−0.05 (61)	−0.09 (63)	−0.12 (53)	−0.20 (61)	−0.20 (62)	−0.18 (64)
Organization of Materials	0.12 (62)	0.01 (64)	−0.08 (54)	0.06 (62)	−0.09 (63)	−0.05 (65)
**Index Scales and Composite Score**						
BRI	0.05 (62)	0.01 (64)	−0.12 (54)	−0.07 (62)	−0.04 (63)	−0.03 (65)
ERI	0.12 (60)	0.04 (62)	0.21 (52)	0.06 (60)	−0.06 (61)	0.08 (63)
CRI	0.10 (61)	−0.03 (63)	−0.01 (53)	−0.08 (61)	−0.18 (62)	−0.13 (64)
GEC	0.16 (61)	0.03 (63)	0.10 (53)	−0.05 (61)	−0.14 (62)	−0.08 (64)

Note. BRI = Behavior Regulation Index; CRI = Cognitive Regulation Index; ERI = Emotion Regulation Index; GEC = Global Executive Composite; MPS = Math Problem Solving; NO = Numerical Operations; PD = Pseudoword Decoding; RC = Reading Comprehension; WIAT-III = Wechsler Individual Achievement Test, Third Edition; WR = Word Reading.

**Table 5:** Correlations between BRIEF2 educator ratings and academic achievement.

	WIAT–III *r* (*n*)					
	PD	WR	RC	Spelling	MPS	NO
**Clinical Scales**						
Inhibit	−0.24 (62)	−0.24 (64)	−0.22 (54)	−0.25 *^a^ (62)	−0.07 (63)	−0.13 (65)
Self-Monitor	−0.17 (62)	−0.22 (64)	−0.18 (54)	−0.18 (62)	−0.12 (63)	−0.16 (65)
Shift	−0.17 (62)	−0.19 (64)	−0.18 (54)	−0.18 (62)	−0.02 (63)	−0.14 (65)
Emotional Control	−0.13 (62)	−0.13 (64)	0.05 (54)	−0.11 (62)	0.001 (63)	−0.10 (65)
Initiate	−0.13 (62)	−0.22 (64)	−0.35 **^a^ (54)	−0.17 (62)	−0.19 (63)	−0.23 (65)
Working Memory	−0.29 *^a^ (62)	−0.37 ** (64)	−0.47 ** (54)	−0.35 ** (62)	−0.24 (63)	−0.30 *^a^ (65)
Plan/Organize	−0.25 (62)	−0.32 ** (64)	−0.36 **^a^ (54)	−0.29 *^a^ (62)	−0.20 (63)	−0.22 (65)
Task–Monitor	−0.30 *^a^ (61)	−0.36 ** (63)	−0.39 ** (53)	−0.39 ** (61)	−0.24 (62)	−0.27 *^a^ (64)
Organization of Materials	−0.14 (62)	−0.22 (64)	−0.32 *^a^ (54)	−0.22 (62)	−0.21 (63)	−0.25 *^a^ (65)
**Index Scales and Composite Score**						
BRI	−0.24 (61)	−0.26 *^a^ (63)	−0.20 (53)	−0.27 *^a^ (61)	−0.04 (62)	−0.12 (64)
ERI	−0.19 (61)	−0.20 (63)	−0.07 (53)	−0.18 (61)	−0.02 (62)	−0.14 (64)
CRI	−0.27 *^a^ (62)	−0.38 ** (64)	−0.45 ** (54)	−0.36 ** (62)	−0.26 *^a^ (63)	−0.29 *^a^ (65)
GEC	−0.25 *^a^ (62)	−0.33 ** (64)	−0.33 *^a^ (54)	−0.31 *^a^ (62)	−0.18 (63)	−0.23 (65)

Note. BRI = Behavior Regulation Index; CRI = Cognitive Regulation Index; ERI = Emotion Regulation Index; GEC = Global Executive Composite; MPS = Math Problem Solving; NO = Numerical Operations; PD = Pseudoword Decoding; RC = Reading Comprehension; WIAT-III = Wechsler Individual Achievement Test, Third Edition; WIAT-III = Wechsler Individual Achievement Test, Third Edition. * *p* < 0.05. ** *p* < 0.01. ^a^ No longer significant after Bonferroni corrections with adjusted critical *p*-values.

**Table 6:** Correlations between D-KEFS scores and academic achievement.

	WIAT–III *r* (*n*)					
	PD	WR	RC	Spelling	MPS	NO
**Tests of Inhibition**						
CWIT I: CT	0.30 *^a^ (60)	0.10 (61)	0.29 *^a^ (53)	0.30 *^a^ (57)	0.29 *^a^ (59)	0.30 *^a^ (60)
CWIT I: TE	0.28 *^a^ (56)	0.10 (57)	0.27 (51)	0.26 (54)	0.28 *^a^ (56)	0.20 (56)
CWIT I/S: CT	0.46 *** (59)	0.38 ** (59)	0.48 *** (52)	0.41 ** (56)	0.43 ** (57)	0.36 (58)
CWIT I/S: TE	0.28 *^a^ (55)	0.18 (55)	0.19 (50)	0.22 (53)	0.28 *^a^ (54)	0.03 (54)
**Tests of Initiation**						
VFT LF: TC	0.37 ** (62)	0.43 ** (62)	0.34 * (54)	0.41 ** (60)	0.17 (61)	0.27 *^a^ (62)
VFT CF: TC	0.06 (61)	0.09 (62)	0.19 (54)	0.20 (59)	0.24 (62)	0.11 (62)
VFT CS: TC	0.23 (60)	0.29 *^a^ (61)	0.32 *^a^ (52)	0.34 ** (58)	0.35 ** (60)	0.40 ** (61)
DF TAD	0.49 ** (33)	0.28 (35)	0.40 *^a^ (29)	0.45 ** (33)	−0.02 (36)	0.21 (35)
**Tests of Self-Monitoring**						
VFT RE	0.07 (59)	0.09 (59)	0.13 (52)	0.13 (57)	−0.03 (59)	0.02 (59)
VFT Percent RE	0.11 (59)	0.14 (59)	0.15 (52)	0.16 (57)	−0.02 (59)	0.05 (59)
DF TRD	−0.16 (34)	−0.23 (36)	−0.22 (30)	−0.20 (35)	0.23 (37)	0.08 (37)
**Tests of Shifting/Switching**						
VFT CS: Total SA	0.29 *^a^ (61)	0.26 *^a^ (62)	0.33 *^a^ (53)	0.24 (59)	0.35 ** (61)	0.28 *^a^ (62)
VFT CS: Percent SA	0.18 (58)	0.24 (59)	0.26 (50)	0.18 (56)	0.31 *^a^ (58)	0.19 (59)
CWIT I/S: CT	0.46 *** (59)	0.38 ** (59)	0.48 *** (52)	0.41 ** (56)	0.43 ** (57)	0.36 (58)
CWIT I/S: TE	0.28 *^a^ (55)	0.18 (55)	0.19 (50)	0.22 (53)	0.28 *^a^ (54)	0.03 (54)
DF Switching: TC	0.34 (34)	0.25 (36)	0.35 (30)	0.27 (35)	0.30 (37)	0.43 ** (37)
**Tests of Task-Monitoring**						
VFT SLE	0.17 (59)	0.19 (59)	0.18 (52)	0.21 (57)	0.13 (59)	0.30 *^a^ (59)
VFT Percent SLE	0.22 (59)	0.25 (59)	0.20 (52)	0.24 (57)	0.09 (59)	0.26 *^a^ (59)
DF TSD	−0.06 (34)	0.06 (36)	0.003 (30)	−0.04 (35)	0.21 (37)	0.28 (37)
**Tests of Working Memory**						
Digit Span	0.35 * (53)	0.30 * (55)	0.51 *** (48)	0.33 * (53)	0.28 *^a^ (54)	0.42 ** (56)
Picture Span	0.16 (44)	0.27 (46)	0.37 * (39)	0.30 *^a^ (45)	0.50 *** (46)	0.42 ** (48)

Note. CT = Completion Time; CWIT I = Color-Word Interference Test Inhibition; CWIT I/S = Color-Word Interference Test Inhibition/Switching; DF = Design Fluency; MPS = Math Problem Solving; NO = Numerical Operations; PD = Pseudoword Decoding; RC = Reading Comprehension; RE = Repetition Errors; SA = Switching Accuracy; SLE = Set-Loss Errors; TAD = Total Attempted Designs; TC = Total Correct; TE = Total Errors; TRD = Total Repeated Designs; TSD = Total Set-Loss Designs; VFT CF = Verbal Fluency Test Category Fluency; VFT CS = Verbal Fluency Test Category Switching; VFT LF = Verbal Fluency Test Letter Fluency; WR = Word Reading. * *p* < 0.05. ** *p* < 0.01. *** *p* < 0.001. ^a^ No longer significant after Bonferroni corrections with adjusted critical *p*-values.

## Data Availability

The data supporting this study’s findings are available on request from the corresponding author. The data are not publicly available due to privacy or ethical restrictions.

## Supplementary Materials

The following supporting information can be downloaded at: https://www.mdpi.com/article/10.3390/children12070842/s1, Table S1: Descriptive statistics for BRIEF2 caregiver and educator ratings for the total sample; Table S2: Mean *T*-Scores and ranges for BRIEF2 caregiver ratings; Table S3: Mean *T*-Scores and ranges for BRIEF2 educator ratings; Table S4: Correlations between direct measures of executive functioning and BRIEF2 index scales and composite scores.
