# Supplementary material for: The Correspondence Between Executive Functioning and Academic Achievement Among Children with Prenatal Alcohol Exposure
Source: Children (Basel). 2025 Jun 26;12(7):842. doi: 10.3390/children12070842 (PMC12293274; doi:10.3390/children12070842)
Supplement: Supplementary file 1 [file children-12-00842-s001.zip › children-3629314-supplementary.docx]

Supplemental Table S1

*Descriptive Statistics BRIEF2 Caregiver and Educator Ratings for the Total Sample*

|  | Total Sample – Caregiver | | |  | Total Sample – Educator | | |
| --- | --- | --- | --- | --- | --- | --- | --- |
|  | Mean (*SD*) | Range | % CE |  | Mean (*SD*) | Range | % CE |
| Clinical Scales |  |  |  |  |  |  |  |
| Inhibit | 67.78 (11.46) | 42–89 | 41.89 |  | 60.86 (13.46) | 41–89 | 28.38 |
| Self-Monitor | 67.42 (9.65) | 49–80 | 43.24 |  | 63.74 (10.78) | 43–84 | 36.49 |
| Shift | 69.89 (10.93)^a^ | 46–91 | 50.00 |  | 67.18 (13.00) | 41–91 | 39.19 |
| Emotional Control | 65.30 (11.95) | 40–84 | 39.19 |  | 63.22 (15.68) | 44–91 | 33.78 |
| Initiate | 68.34 (7.71) | 43–84 | 55.41 |  | 66.27 (10.98) | 40–89 | 40.54 |
| Working Memory | 71.34 (9.19) | 44–87 | 64.86 |  | 70.76 (12.28) | 43–91 | 54.05 |
| Plan/Organize | 66.28 (8.47) | 40–80 | 37.84 |  | 66.35 (11.66) | 41–89 | 43.24 |
| Task-Monitor | 62.04 (8.90)^a^ | 40–77 | 17.57 |  | 63.81 (11.24)^a^ | 41–86 | 41.89 |
| Organization of Materials | 63.91 (8.33) | 46–78 | 29.73 |  | 65.16 (11.28) | 44–86 | 35.14 |
| Index Scales and Composite Score |  |  |  |  |  |  |  |
| **Behavior Regulation Index** | 68.81 (10.83) | 44–89 | 52.70 |  | 62.55 (11.83)^a^ | 42–84 | 29.73 |
| **Emotion Regulation Index** | 68.82 (11.30)^b^ | 44–90 | 43.24 |  | 66.04 (13.97)^a^ | 43–91 | 40.54 |
| **Cognitive Regulation Index** | 69.21 (7.72)^a^ | 42–81 | 59.46 |  | 68.81 (11.32) | 44–91 | 54.05 |
| Global Executive Composite | 71.90 (9.04)^a^ | 44–87 | 64.86 |  | 68.46 (11.59) | 47–88 | 51.35 |
| Validity Scales (*N/Total N*) |  |  |  |  |  |  |  |
| Negativity | Acceptable | Elevated | Highly Elevated |  | Acceptable | Elevated | Highly Elevated |
|  | 67/68 | 0/68 | 1/68 |  | 65/67 | 2/67 | 0/67 |
|  | Acceptable | Questionable |  |  | Acceptable | Questionable |  |
| Inconsistency | 66/69 | 3/69 |  |  | 66/67 | 1/67 |  |
| Infrequency^c^ | 58/69 | 11/69 |  |  | 65/67 | 2/67 |  |

*Note.* N = 74 unless otherwise noted. ^a^n = 73, ^b^n = 72. Some of the validity scales were missing. 67/68 means that 67 of the 68 caregiver forms with the Negativity Scale ratings were classified as Acceptable. ^c^The Questionable Infrequency Scale classification should be interpreted cautiously because the endorsed items sometimes reflected accurate functioning.

Supplemental Table S2

*Mean T-Scores and Ranges for BRIEF2 Caregiver Ratings*

|  | PAE with FASD | | |  | PAE without FASD | | |  |  |
| --- | --- | --- | --- | --- | --- | --- | --- | --- | --- |
|  | Mean (*SD*) | Range | % CE |  | Mean (*SD*) | Range | % CE |  | *t* |
| Clinical Scales |  |  |  |  |  |  |  |  |  |
| Inhibit | 70.12 (11.35) | 42–89 | 46.5 |  | 64.55 (11.00)^a^ | 43–89 | 35.5 |  | –2.11* |
| Self-Monitor | 68.28 (9.54) | 49–80 | 46.5 |  | 66.23 (9.83)^a^ | 49–80 | 38.7 |  | –0.90 |
| Shift | 70.33 (10.75) | 46–91 | 51.2 |  | 69.27 (11.35)^b^ | 46–91 | 48.4 |  | –0.40 |
| Emotional Control | 66.00 (11.84) | 40–84 | 41.9 |  | 64.32 (12.23)^a^ | 41–84 | 35.5 |  | –0.59 |
| Initiate | 68.35 (7.48) | 50–79 | 58.1 |  | 68.32 (8.13)^a^ | 43–84 | 51.6 |  | –0.01 |
| Working Memory | 71.30 (8.45) | 49–87 | 62.8 |  | 71.39 (10.29)^a^ | 44–83 | 67.7 |  | 0.04 |
| Plan/Organize | 66.81 (8.35) | 45–80 | 37.2 |  | 65.55 (8.72)^a^ | 40–80 | 38.7 |  | –0.63 |
| Task-Monitor | 63.77 (8.67) | 40–77 | 23.3 |  | 59.57 (8.78)^b^ | 41–74 | 9.7 |  | –2.03* |
| Organization of Materials | 64.74 (7.06) | 48–77 | 27.9 |  | 62.74 (9.85)^a^ | 46–78 | 32.3 |  | –0.97 |
| Index Scales and Composite Score |  |  |  |  |  |  |  |  |  |
| **Behavior Regulation Index** | 70.56 (11.17) | 44–89 | 62.8 |  | 66.39 (10.01)^a^ | 45–82 | 38.7 |  | –1.65 |
| **Emotion Regulation Index** | 69.44 (11.29) | 47–90 | 44.2 |  | 67.90 (11.45)^c^ | 44–89 | 41.9 |  | –0.57 |
| **Cognitive Regulation Index** | 69.77 (7.08) | 49–81 | 65.1 |  | 68.40 (8.61)^b^ | 42–79 | 51.6 |  | –0.74 |
| Global Executive Composite | 72.86 (8.82) | 48–87 | 69.8 |  | 70.53 (9.33)^b^ | 44–85 | 58.1 |  | –1.08 |

*Note.* % CE = Percentage of children with ratings falling in the clinically elevated range (*T*-scores > 70); FASD = fetal alcohol spectrum disorder; PAE = prenatal alcohol exposure.

* *p* < .05. ** *p* <.01. *** *p* <.001.

*Note.* N = 43 unless otherwise noted. ^a^n = 31, ^b^n = 30 , ^c^n = 29.

Supplemental Table S3

*Mean T-Scores and Ranges for BRIEF2 Educator Ratings*

|  | PAE with FASD | | |  | PAE without FASD | | |  |  |
| --- | --- | --- | --- | --- | --- | --- | --- | --- | --- |
|  | Mean (*SD*) | Range | % CE |  | Mean (*SD*) | Range | % CE |  | *t* |
| **Clinical Scales** |  |  |  |  |  |  |  |  |  |
| Inhibit | 62.93 (14.53) | 41–89 | 37.2 |  | 58.00 (11.45)^b^ | 43–86 | 16.1 |  | –1.63 |
| Self-Monitor | 64.58 (10.83) | 43–82 | 39.5 |  | 62.58 (10.78)^b^ | 43–84 | 32.3 |  | –0.79 |
| Shift | 69.67 (12.95) | 41–91 | 51.2 |  | 63.71 (12.45)^b^ | 42–91 | 22.6 |  | –1.99 |
| Emotional Control | 64.86 (15.90) | 44–91 | 34.9 |  | 60.94 (15.34)^b^ | 44–91 | 32.3 |  | –1.06 |
| Initiate | 67.09 (9.60) | 45.84 | 44.2 |  | 65.13 (12.74)^b^ | 40–89 | 35.5 |  | –0.76 |
| Working Memory | 72.23 (10.87) | 43–90 | 60.5 |  | 68.71 (13.94)^b^ | 43–91 | 45.2 |  | –1.22 |
| Plan/Organize | 66.74 (10.96) | 44–86 | 44.2 |  | 65.81 (12.73)^b^ | 41–89 | 41.9 |  | –0.34 |
| Task-Monitor | 64.91 (10.87) | 42–82 | 46.5 |  | 62.23 (11.76)^c^ | 41–86 | 35.5 |  | –1.00 |
| Organization of Materials | 66.37 (11.08) | 46–82 | 41.9 |  | 63.48 (11.52)^b^ | 44–86 | 25.8 |  | –1.09 |
| **Index Scales and Composite Score** |  |  |  |  |  |  |  |  |  |
| BRI | 63.95 (12.64)^a^ | 42–84 | 34.9 |  | 60.65 (10.53)^b^ | 46–83 | 22.6 |  | –1.18 |
| ERI | 68.40 (13.95) | 44–91 | 46.5 |  | 62.67 (13.52)^c^ | 43–91 | 32.3 |  | –1.75 |
| CRI | 69.91 (10.37) | 44–85 | 60.5 |  | 67.29 (12.53)^b^ | 46–91 | 45.2 |  | –0.98 |
| GEC | 70.16 (11.30) | 47–87 | 62.8 |  | 66.10 (11.75)^b^ | 49–88 | 35.5 |  | –1.50 |

*Note.* N = 43 unless otherwise noted. ^a^n = 42, ^b^n = 31, ^c^n = 30. BRI = **Behavior Regulation Index; CRI = Cognitive Regulation Index; ERI = Emotion Regulation Index; GEC =** Global Executive Composite.

Supplemental Table S4

*Correlations between Direct Measures of Executive Functioning and BRIEF2 Index Scales and Composite Scores*

|  | **BRIEF2** | **Caregiver** | **Educator** |
| --- | --- | --- | --- |
| **Tests of Inhibition** |  |  |  |
| CWIT Inhibition: Completion Time | BRI | .18 (69) | –.06 (68) |
|  | ERI | .15 (67) | –.01 (68) |
|  | CRI | .10 (68) | –.01 (69) |
|  | GEC | .15 (68) | –.01 (69) |
| CWIT Inhibition: Total Errors | BRI | –.17 (65) | –.31 (64)* |
|  | ERI | .04 (63) | –.14 (64) |
|  | CRI | –.04 (64) | –.04 (65) |
|  | GEC | –.04 (64) | –.15 (65) |
| CWIT Inhibition/Switching: Completion Time | BRI | .16 (67) | .04 (66) |
|  | ERI | .12 (65) | .01 (66) |
|  | CRI | .11 (66) | –.10 (67) |
|  | GEC | .20 (66) | –.02 (67) |
| CWIT Inhibition/Switching: Total Errors | BRI | –.23 (63) | –.31 (62)* |
|  | ERI | –.004 (61) | –.19 (62) |
|  | CRI | .006 (62) | –.01 (63) |
|  | GEC | –.03 (62) | –.16 (63) |
| **Tests of Initiation** |  |  |  |
| VFT Letter Fluency: Total Correct | BRI | .21 (71) | –.23 (70) |
|  | ERI | .07 (69) | –.23 (70) |
|  | CRI | .12 (70) | –.30 (71)* |
|  | GEC | .11 (70) | –.29 (71)* |
| VFT Category Fluency: Total Correct | BRI | –.02 (71) | –.18 (70) |
|  | ERI | –.06 (69) | –.13 (70) |
|  | CRI | .03 (70) | –.18 (71) |
|  | GEC | –.07 (70) | –.18 (71) |
| VFT Category Switching: Total Correct Responses | BRI | –.14 (70) | –.27 (69)* |
|  | ERI | –.18 (68) | –.19 (69) |
|  | CRI | –.04 (69) | –.15 (70) |
|  | GEC | –.20 (69) | –.21 (70) |
| DF Total Attempted Designs | BRI | –.04 (42) | –.05 (42) |
|  | ERI | .01 (40) | –.02 (41) |
|  | CRI | .05 (41) | –.23 (42) |
|  | GEC | .05 (41) | –.12 (42) |
| **Tests of Self-Monitoring** |  |  |  |
| VFT Repetition Errors | BRI | –.01 (68) | –.19 (67) |
|  | ERI | .12 (66) | –.03 (67) |
|  | CRI | –.01 (67) | –.18 (68) |
|  | GEC | –.02 (67) | –.19 (68) |
| VFT Percent Repetition Errors | BRI | .03 (68) | –.25 (67)* |
|  | ERI | .10 (66) | –.11 (67) |
|  | CRI | .04 (67) | –.21 (68) |
|  | GEC | .02 (67) | –.24 (68) |
| DF Total Repeated Designs | BRI | –.18 (44) | –.10 (44) |
|  | ERI | –.16 (42) | –.11 (43) |
|  | CRI | –.13 (43) | .13 (43) |
|  | GEC | –.24 (43) | –.02 (44) |
| **Tests of Shifting/Switching** |  |  |  |
| VFT Category Switching: Total Switching Accuracy | BRI | –.10 (71) | 0.27 (70)* |
|  | ERI | –.11 (69) | –.16 (70) |
|  | CRI | .07 (70) | –.16 (71) |
|  | GEC | –.08 (70) | –.21 (71) |
| VFT Category Switching: Percent Switching Accuracy | BRI | –.19 (68) | –.22 (67) |
|  | ERI | –.09 (66) | –.08 (67) |
|  | CRI | –.04 (67) | –.12 (68) |
|  | GEC | –.15 (67) | –.16 (68) |
| CWIT Inhibition/Switching: Completion Time | BRI | .16 (67) | .04 (66) |
|  | ERI | .12 (65) | .01 (66) |
|  | CRI | .11 (66) | –.10 (67) |
|  | GEC | .20 (66) | –.02 (67) |
| CWIT Inhibition/Switching: Total Errors | BRI | –.23 (63) | –.31 (62)* |
|  | ERI | –.004 (61) | –.19 (62) |
|  | CRI | .006 (62) | –.01 (63) |
|  | GEC | –.03 (62) | –.16 (63) |
| **Tests of Task-Monitoring** |  |  |  |
| VFT Set-Loss Errors | BRI | –.20 (68) | –.18 (67) |
|  | ERI | .05 (66) | –.05 (67) |
|  | CRI | –.09 (67) | –.07 (68) |
|  | GEC | –.12 (67) | –.11 (68) |
| VFT Percent Set-Loss Errors | BRI | –.17 (68) | –.26 (67)* |
|  | ERI | .02 (66) | –.14 (67) |
|  | CRI | –.05 (67) | –.13 (68) |
|  | GEC | –.10 (67) | –.19 (68) |
| DF Total Set-Loss Designs | BRI | –.40 (44)** | –.24 (44) |
|  | ERI | –.32 (42)* | –.21 (43) |
|  | CRI | –.28 (43) | –.14 (44) |
|  | GEC | –.42 (43)** | –.21 (44) |
| **Tests of Working Memory** |  |  |  |
| Digit Span | BRI | –.19 (57) | –.34 (56)** |
|  | ERI | –.27 (55)* | –.31 (56)* |
|  | CRI | .15 (56) | –.16 (57) |
|  | GEC | –.07 (56) | –.28 (57)* |
| Picture Span | BRI | –.22 (49) | –.26 (48) |
|  | ERI | –.19 (47) | –.26 (48) |
|  | CRI | –.13 (48) | –.30 (49)* |
|  | GEC | –.20 (48) | –.33 (49)* |

*Note.* Sample sizes are provided in brackets. BRI = Behavior Regulation Index; CRI = Cognitive Regulation Index; CWIT = Color-Word Interference Test; DF = Design Fluency; ERI = **Emotion Regulation Index;** GEC = Global Executive Composite; VFT = Verbal Fluency Test. * *p* < .05. ** *p* <.01. *** *p* <.001.
